# Molecular typing and characterization of a novel genotype of EV-B93 isolated from Tibet, China

**DOI:** 10.1371/journal.pone.0237652

**Published:** 2020-08-25

**Authors:** Man Zhang, Yong Zhang, Mei Hong, Jinbo Xiao, Zhenzhi Han, Yang Song, Shuangli Zhu, Dongmei Yan, Qian Yang, Wenbo Xu, Zhijun Liu

**Affiliations:** 1 Department of Medical Microbiology, Weifang Medical University, Weifang, People’s Republic of China; 2 WHO WPRO Regional Polio Reference Laboratory and National Health Commission Key Laboratory for Biosafety, National Institute for Viral Disease Control and Prevention, Chinese Center for Disease Control and Prevention, Beijing, People’s Republic of China; 3 Center for Biosafety Mega-Science, Chinese Academy of Sciences, Beijing, People’s Republic of China; 4 Tibet Center for Disease Control and Prevention, Lhasa City, Tibet Autonomous Region, People’s Republic of China; Institut Pasteur, FRANCE

## Abstract

EV-B93 is a novel serotype within the *Enterovirus B* species and is uncommon worldwide. Currently, only one full-length genomic sequence (the prototype strain) has been deposited in the GenBank database. In this study, three EV-B93 were identified, including one from an acute flaccid paralysis (AFP) patient (named 99052/XZ/CHN/1999, hereafter XZ99052) and two from healthy children (named 99096/XZ/CHN/1999 and 99167/XZ/CHN/1999, hereafter XZ99096 and XZ99167, respectively) from Tibet in 1999 during the polio eradication program. The identity between the nucleotide and amino acid sequences of the Tibet EV-B93 strain and the EV-B93 prototype strain is 83.2%–83.4% and 96.8%–96.9%, respectively. The Tibet EV-B93 strain was found to have greater nucleotide sequence identity in the *P3* region to another enterovirus EV-B107 as per a phylogenetic tree analysis, which revealed that recombination occurred. Seroepidemiology data showed that EV-B93 has not produced an epidemic in Tibet and there may be susceptible individuals. The three Tibet EV-B93 strains are temperature-resistant with prognosticative virulence, suggesting the possibility of a potential large-scale outbreak of EV-B93. The analyzed EV-B93 strains enrich our knowledge about this serotype and provide valuable information on global EV-B93 molecular epidemiology. What is more, they permit the appraisal of the serotype's potential public health impact and aid in understanding the role of recombination events in the evolution of enteroviruses.

## Introduction

Enteroviruses belong to the order *Picornavirales* of the *Picornavirus* family, which comprises 15 species designated as *Enterovirus A–H* and *J*, *Rhinovirus A–C*; species *Enterovirus A–D* contain more than 100 serotypes, such as poliovirus, coxsackievirus, echovirus, and recently discovered enteroviruses [1–4]. These viruses are single-stranded positive-sense RNA viruses with a genome size of about 7400–7500 bps and consist of a large open reading frame (ORF) flanked by 5' and 3' untranslated regions (UTR) [5–7]. The 5′-UTR is about 740 nucleotides long and has an internal ribosome entry site (IRES) that is indispensable for translation initiation and comprises several structured RNA domains denoted II to VI [8]. The polyprotein ORF for protein synthesis is split in three parts: *P1*, *P2*, and *P3*; where *P1* encodes the structural proteins VP1, VP2, VP3, and VP4 of the virus. Among them, VP1 is often used for enterovirus typing due to contains many epitopes and is highly conserved. *P2* and *P3* encode seven non-structural proteins, including 2A, 2B, 2C, 3A, 3B, 3C, and 3D. The four structural proteins VP1-VP4 assemble to form a protomer, and five of such protomers are assembled into subunits of a pentameric structure, and 12 such subunits are interconnected by respective domains to form a viral outer shell [9, 10].

Generally speaking, all enteroviruses contain only one ORF (polyrotein ORF). However, some studies suggest that many enterovirus genomes also contain an upstream ORF (uORF), which is subject to strong purifying selection [11]. Domain VI in IRES comprises a stem-loop containing a highly conserved AUG codon. Sequences were defined as having the uORF if the ORF beginning with this AUG codon overlaps the 5' end of the polyprotein ORF and contains at least 150 nt upstream of the polyprotein AUG codon. Studies have shown that 97.8% of *Enterovirus* A and 85.4% of *Enterovirus* B have uORF [8].

Enteroviruses have complex pathogenesis and can cause a wide spectrum of diseases, but most people get a silent infection. Clinical symptoms of various severities may occur in cases of depressed immunity and recurrent exposure to viruses, such as acute flaccid paralysis (AFP); hand, foot, and mouth disease; aseptic meningitis; acute pulmonary oedema; myocarditis [12–17]. Some critically ill patients may die quickly.

AFP is not a single disease type, but a group of syndromes characterized by an acute onset, decreased muscle tone, decreased muscle strength, and reduction or complete loss of tendon reflexes. Poliovirus is one of the major causes of AFP and belongs to EV-C [18, 19]. Children are prone to acute paralysis syndrome mainly because the neuro-immune system of infants and young children is not yet mature; their immune function is weak and susceptible to viral infection, and their nervous system is still developing. While most AFP patients have a good prognosis, a small number of children children may have cognitive sequelae, such as dyskinesia. Due to the broadened AFP definition and enhanced surveillance, many non-polio AFP cases have been reported and many recently discovered enteroviruses, such as EV-73 [20], EV-99 [21], EV-105 [22] have been identified as causative pathogens.

EV-B93 is a recently discovered enterovirus, and its prototype strain was isolated from the faeces of AFP patients in Congo in 2000 [23]. Currently, only one prototype EV-B93 complete genome sequence is known to have been deposited in GenBank [24]. Among 11 EV-B93 strains with partial or entire *VP1* sequences, 8 were isolated from AFP patients [25–28], 2 were found in the stool of patients with acute gastroenteritis [29, 30], and one was found in healthy children but could not be retrieved from cell culture [31]. The first Chinese EV-B93 reported in 2008, was also isolated from the faeces of AFP patients. In this study, three EV-B93 were isolated from an AFP patient and two healthy children during surveillance studies of AFP conducted in Tibet in China in 1999, and were named XZ99052 (from AFP patient), XZ99096, and XZ99167 respectively. Although the number of EV-B93 is reported to be limited to only 15 strains till now, this may be related to the fact that most enteroviruses are recessive infections. However, based on the above situation, the relationship between EV-B93 and AFP has aroused our vigilance.

## Methods

### Ethics statement and sample collection

This study didn’t involve human participants or experimentation; the materials used in the study were stool samples that were collected during AFP surveillance activities in accordance with the World Health Organization (WHO) guidelines. Written informed consent for the use of stool samples was obtained from their parents involved in this study. All experimental technical and ethical aspects were approved by the Ethics Review Committee of the National Institute for Viral Disease Control and Prevention, Chinese Center for Disease Control and Prevention; and the methods were carried out in accordance with the approved guidelines.

### Sample collection and virus isolation

Three stool samples (XZ99052, XZ99096, and XZ99167) were collected from an AFP patients, and two healthy children in Tibet in 1999; XZ99052 was collected from an AFP case from a two-year-old boy, strain XZ99096 was from a health child of four-month-old girl, and strain XZ99167 was collected from a health child of four-year-old girl. All samples were collected and processed according to standard procedures [32] in 1999 during poliovirus surveillance activities. The processed samples were inoculated into two cell lines, a RD cell and a mouse cell line with the human poliovirus receptor (L20B). Both cell lines were provided by the WHO Global Poliovirus Specialized Laboratory in the US and originally purchased from the American type Culture Collection (Manassas, VA, USA). When the virus EV-like CPE attained phanerosis, we harvested the infected cell cultures. Cytopathic effect was just observed in the RD cell line. For the seroprevalence study of EV-B93 antibodies, 56 healthy children ≤5 years of age were surveyed. Fifty-six serum samples were collected randomly in 2010, with informed parental consent by the Tibet Center for Disease Control and Prevention.

### Molecular typing and full-length genome sequencing of the three EV-B93 strains

Viral RNA was extracted from the cell culture using a QIAamp Viral RNA Mini Kit (Qiagen, Hilden, Germany). The partial *VP1* region was amplified using reverse transcription polymerase chain reaction (RT-PCR) with the primer pairs 490 and 492 [33] by using the PrimeScript One Step RT-PCR Kit Ver.2 (TaKaRa, Dalian, China). The positive products after amplification were purified using the QIAquick PCR purification kit (Qiagen, Germany), and then sequenced in both directions at least once from each strand using ABI 3130 Genetic Analyser (Applied Biosystems, Foster City, CA, USA). The *VP1* sequences obtained were compared to the homologous sequences available in GenBank database using the program BLAST (Basic Local Alignment Search Tool) in NCBI. Serotype was confirmed with the EV Genotyping Tool [34].

The 5′ end and the 3′ end of the genome was amplified based on the manufacturer’s instructions with the 5′-Full RACE Kit (Takara, Shiga, Japan) and an oligo-dT primer (7500A) previously reported in another study [35]. The remained full-length genome sequences of the virus were amplified by the primer walking strategy. Briefly, overlapping fragments representing whole genomes were amplified by RT-PCR using specific primers (Table 1). The RT-PCR products were purified and sequenced as described above.

**Table 1:** Primers used for RT-PCR and full-length genomic sequencing of EV-B93.

Primer	Nucleotide position (nt)	Primer sequence (5’-3’)
0001S48 [35]		GGGGACAAGTTTGTACAAAAAAGCAG
EV-B93-755R	736–755	GGTTTTCTGCGTTGACACCT
EV-B93-968F	968–987	AGTGCGGGTACAGTGATAGG
EV-B93-2051R	2032–2051	GCCTGACCAATGTGCGTAAT
EV-B93-1786F	1786–1765	TACCAGTCACCATCCGCAAT
EV-B93-2607R	2588–2607	AGTTGCACACGTGTCTTGTC
EV-B93-3187F	3187–3206	ATTCCACGACCACCAAGACT
EV-B93-4111R	4092–4111	GCGATCCATTCCATGCCTTT
EV-B93-4070F	4070–4089	ACTGGCCCTCATTGGTTGTA
EV-B93-5005R	4986–5005	ACCCTGTGTCTGTGGTTGTAT
EV-B93-4732F	4732–4851	ACAGGTAAAGTGCTCGGGAT
EV-B93-5603R	5584–5603	CGGATTGAGGTGGAAGGTCT
EV-B93-5943F	5962–5943	AGACCTTCCACCTCAATCCG
EV-B93-6522R	6503–6522	CGGATTGAGGTGGAAGGTCT
EV-B93-6522F	6522–6541	AGACCTTCCACCTCAATCCG
7500A [35]		GGGGACCACTTTGTACAAGAAAGCTGGG (T_24_)

### Phylogenetic and recombination analysis

The nucleotide sequences and deduced amino acid sequences of the Chinese EV-B93 strain were compared with those of the prototype EV-B strains by pairwise alignment using the MEGA program (version 7.0) [36]. The identity matrix was processed using BioEdit (version 7.0.9.0) [37]. Phylogenetic trees were constructed by the neighbour-joining method, implemented in the MEGA program, using the Kimura 2-parameter model. Bootstrapping was performed with 1000 bootstrap replicates and bootstrap values greater than 80% were considered statistically significant for grouping. Identity plots and bootscanning analyses were performed using the SimPlot 3.5.1 program. A 200-nucleotide window was moved in 20-nucleotide steps and bootscanning analyses were operated using the neighbour-joining method [38].

### Neutralization assay against EV-B93

In our study, we use micro-neutralization assay to detect antibodies and evaluate seroprevalence. Strain XZ99052 was picked up as the attack strain by the results that antigenically equivalent (VP1 amino acid identity among the three EV-B93 strains was 290) and the highest TCID_50_ value among the three Tibet EV-B93 strains. Serum sample was inactivated by incubation at 56°C for 30 min. Each sample was serially diluted (1:4 to 1:1024), and aliquots (50 μL) of five different concentrations were placed in duplicate into a 96-well plate. 100 TCID_50_ of a 1:1 admixture of serially diluted serum sample (50 μL): virus (50 μL), were incubated at 36°C in a CO_2_ incubator. Then, we added the same volume of RD cells to each well and two wells in each column were used as serum controls. We also used a new plate for a cell control and virus control that we called a virus back-titration. All the plates were incubated for 7 days. Only when the virus titre in the back-titration plate was between 32 and 320 TCID_50_ and when the cell control was acceptable did we judge the result as effective. Then, the highest dilution of serum where 50% of the cultures were protected from CPE was recorded. The titre was calculated statistically with SAS version 9.4 using Wilcoxon rank sum test, the reason is that when the titer of the sample is lower than 1:4, we hardly collect the lowest serum titer. The dataset contains ranked data, so a rank sum test is helpful to evaluate the difference and data significance. A serum sample was considered positive if the neutralization antibody was observed at a dilution of 1:8, and the GMT of all positive serum samples was calculated.

### Assay of temperature sensitivity

The temperature sensitivity of the three plaque purified EV-B93 strains was evaluated by incubating them on monolayer RD cells with two selected control strains (HTYT-ARL-AFP02F/XJ/CHN/2011, showing non-temperature sensitivity and KS-MGTH90F/XJ/CHN/ 2011, showing temperature sensitivity) in incubators at 36°C and 39.5°C. The plates were harvested at 5 time points post-infection (4, 8, 16, 24, and 48h), and its TCID_50_ was determined. TCID_50_ of a 1:1 admixture of continuous ten-fold diluted virus (100 μL): RD cell (100 μL), incubated at 36°C in a CO_2_ incubator. After repeating this procedure for 4 times for each strain, the TCID_50_ was calculated based on the end-point dilution method on monolayer RD cells in 96-well plates at 36°C. Virus isolates showing more than 2-logarithm reduction in titre at different temperatures were considered temperature-sensitive.

### Data availability and nucleotide sequence accession number

The full-length genomic sequences of the six EV-B93 strains (strain XZ99052, XZ99096, XZ99167) described in this study were uploaded to the GenBank (under the accession numbers MN580134, MN580135, MN580136).

## Results

### Isolation and molecular typing of the three Tibet EV-B93 isolates

The three stool samples were inoculated into rhabdomyoblastoma (RD) cells; they were harvested when the cytopathic effects with enteroviral characteristics appeared. Positive isolates of RD cell were then passed into L20B cells (containing poliovirus receptor, sensitive to poliovirus), and no cytopathic effect (CPE) was observed. The initial judgment was non-polio enterovirus (NPEV). The partial *VP1* region was amplified using species-specific primers 490/492, and the serotype was confirmed by nucleotide sequencing of *VP1* region [39]. After blast alignment using an online EV genotyping tool [40], the identity with the EV-B93 prototype strain was found to be 98%, and it was identified as EV-B93 (Table 2).

**Table 2:** VP1 nucleotide identities between the three EV-B93 strains and prototype strains of the EV-A, EV-B, EV-C, EV-D species.

Strains	EV-B93-38-03 (prototype)	Species A	Species B	Species C	Species D
XZ99052	88.7	37.5–45.3	55.8–66.3	41.6–45.2	42.3–44.4
XZ99096	88.2	37.3–45.2	55.5–66.2	41.8–45.0	42.4–44.0
XZ99167	88.6	37.5–45.0	55.9–66.2	41.6–44.9	42.3–44.5

### Full-length genomic analysis of the three EV-B93 strains

Full-length genome sequences of the three Tibet EV-B93 strains were obtained using the primer walking strategy. The nucleotide sequences and the deduced amino acid sequences of the three Tibet EV-B93 were compared against those of the EV-B93 prototype strains and EV-B serotypes (Table 3); and genomic nucleotide and deduced amino acid identity of 83.2%–83.4% and 96.8%–96.9%, respectively was observed.

**Table 3:** Pairwise nucleotide and amino acid sequence identities between the three EV-B93 strains and prototype strains of the EV-B species.

Region	Nucleotide identity (%) [Amino acid identity (%)]					
	Strain XZ99052		Strain XZ99096		Strain XZ99167	
	Prototype of EV-B93	Prototypes of other EV-B	Prototype of EV-B93	Prototypes of other EV-B	Prototype of EV-B93	Prototypes of other EV-B
5'-UTR	92	66.2–88.4	90.2	64.4–86.6	91.4	65.7–87.8
*VP4*	91.7(98.5)	67.1–79.7(73.9–89.8)	90.8(98.5)	66.6–79.2(73.9–89.8)	91.7(98.5)	67.1–79.7(73.9–89.8)
*VP2*	88.8(97.7)	63.4–71.6(73–83.8)	87.7(96)	61.1–68.6(70.1–80.5)	87.3(95.6)	60.7–67.8(69.8–80.2)
*VP3*	89.4(97.4)	63.6–71.5(68.6–80.3)	89.8(97.9)	63.8–71.7(69–80.7)	89.4(97)	63.6–71.5(68.6–79.9)
*VP1*	88.7(96.5)	55.4–67.5(55.2–70.7)	88.2(95.8)	55.2–67.3(55.5–71.1)	88.6(96.5)	55.5–67.3(55.2–71.4)
*2A*	80.6(94)	75.1–81.5(89.3–96)	81.3(94)	75.5–81.3(89.3–96)	80.6(93.3)	75.5–81.1(89.3–96)
*2B*	79.1(97.9)	77.4–88.5(94.9–100)	79.1(97.9)	77.4–88.5(94.9–100)	78.4(96.9)	70.0–80.8(93.9–98.8)
*2C*	81.9(96)	78.2–87.3(95.7–98.4)	81.8(96.3)	78.3–87.4(95.4–98.7)	82(96.6)	78.4–87.1(95.7–98.4)
*3A*	81.6(97.7)	75.6–88.0(92.1–100)	81.6(97.7)	75.6–88.0(92.1–100)	82(97.7)	75.6–87.6(92.1–100)
*3B*	81.8(95.4)	74.2–89.3(86.3–100)	81.8(95.4)	74.2–89.3(86.3–100)	81.8(95.4)	74.2–89.3(86.3–100)
*3C*	77(96.7)	75.5–85.0(92.3–98.3)	77(96.7)	75.5–85.2(92.3–98.3)	77(96.7)	75.2–84.8(92.3–98.3)
*3D*	78.8(96.9)	76.9–91.1(95.2–99.3)	78.7(96.7)	77.0–91.1(95–99.1)	78.8(97.1)	76.9–91.1(95.4–99.5)
3'-UTR	N/A	77.8–93.2	N/A	77.8–93.2	N/A	77.8–93.2

All the three Tibetan EV-B93 strains were 7438 bp in length, with a polyprotein ORF of 6576 nucleotides each, encoding 2192 amino acids. The overall base composition of the three Tibetan EV-B93 strains was 28% of A, 24% of C, 23% of G, and 25% of U. The genome of the Tibetan EV-B93 isolates comprised 1117 mutations as compared to the EV-B93 prototype strain, with 1039 synonymous changes and 78 non-synonymous substitutions. The non-coding region 5’UTR and 3’UTR consist of 759 and 102 nucleotides, respectively. The three EV-B93 shared 98.6%–99.2% nucleotide and 99.4%–99.6% amino acid similarities with each other. Base composition and homology analysis showed that the three strains were all from the same clone strain.

**Fig 1:**
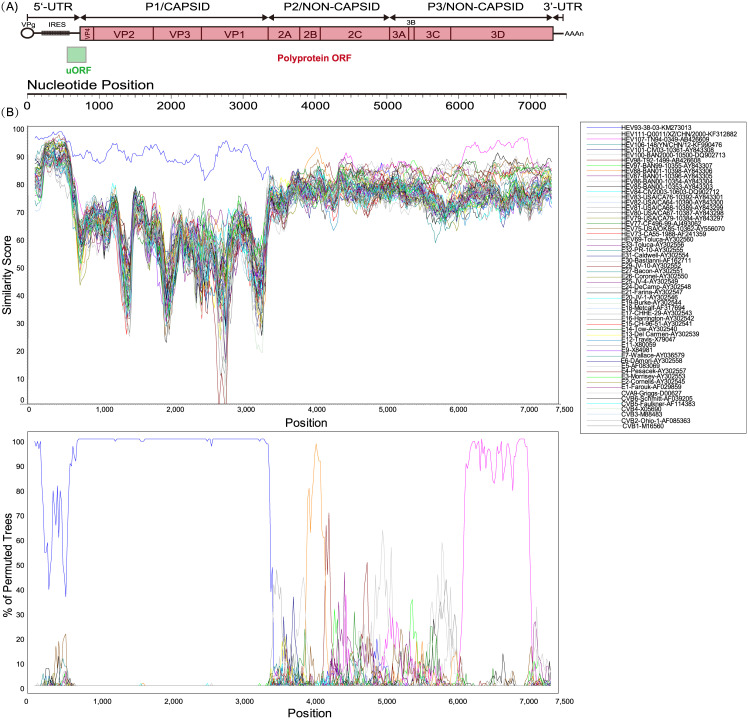
Similarity plot and bootscanning analyses of Chinese EV-B93 strains and other EV-B prototype strains. (A). Genetic organization of Chinese EV-B93 strains. The polyprotein ORF, flanked by 5′-UTR and 3′-UTR, is indicated in red; the upstream ORF, a 68-codon protein that overlaps the polyprotein ORF by 48 nt, is indicated in green. (B). Similarity plot and bootscanning analysis. A sliding window of 200 nucleotides was used, moving in 20-nucleotide steps. The Chinese EV-B93 strain XZ99052/XZ/CHN/1999 was used as a query sequence.

In addition to polyprotein ORF, uORF was found in all three Tibetan EV-B93 sequences. The AUG codon in domain VI AUG of the IRES was identified based on the conserved sequences surrounding it (typically UU **AUG** GUG ACA). The domain VI AUG in IRES (nt591) in Tibet EV-B93 is followed by a 68-codon uORF that overlaps the polyprotein ORF by 48 nt (Fig 1). However, its expression as a protein remains to be confirmed.

### Phylogenetic analysis of global EV-B93 and other EV-B

**Fig 2:**
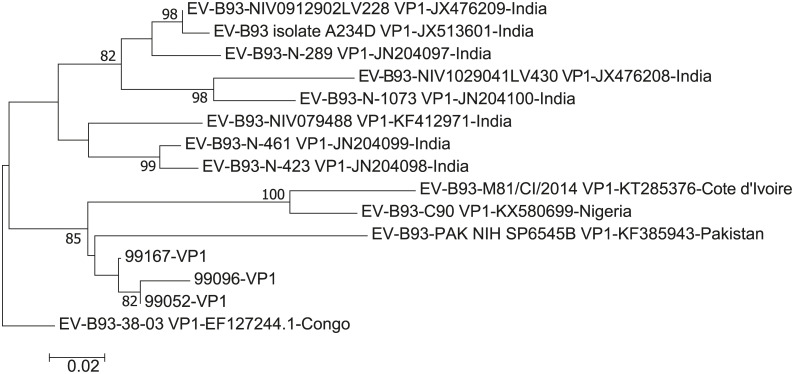
Phylogenetic tree based on the 245-nt *VP1* sequences of Chinese EV-B93 and other EV-B93 strains available in GenBank.

A total of 12 *VP1* sequences of EV-B93 were obtained from GenBank. Out of these 12 EV-B93, most (eight) were isolated in India; the remaining four were isolated in Congo, Côte d'Ivoire, Nigeria, and Pakistan [23–31]. A phylogenetic tree based on 245-nucleotides of the *VP1* sequence of EV-B93 was drawn (Fig 2). The three Tibetan EV-B93 strains showed the highest nucleotide identity (88.4%-89.2%) with the Pakistan strains on the analyzed *VP1* region. However, no clear clusters can be seen in the tree because of the low number of sequences available in GenBank and the small size of some of them.

In order to investigate the similarities in genetic characteristics of EV-B93 and other EV-B strains, phylogenetic trees based on the entire *VP1*, *P1*, *P2*, and *P3* coding regions were performed among the three Tibet EV-B93 and EV-B prototypes; the results showed that the three Tibet EV-B93 were always clustered together, confirming that the three strains are closely related. Interestingly, the Tibet EV-B93 clustered with EV-B93 prototype strain only in VP1 and P1 tree, and they were separated in the *P2* and *P3* tree. Through nucleotide identity analysis, they were clustered together with echovirus 6(97.1%) in the *P2* region and EV-B107 (88.8%) in the *P3* region, suggesting that recombination events might have occurred.

### Recombinant structure of the Chinese EV-B93 strains

Recombination often occurs between different serotypes or genotypes of enteroviruses, with the probability of recombination events being positively correlated with nucleotide identity. It is well known that enteroviruses show higher nucleotide similarities within species than inter-species; and the *P2* and *P3* regions are highly susceptible to recombination within species [41, 42].

**Fig 3:**
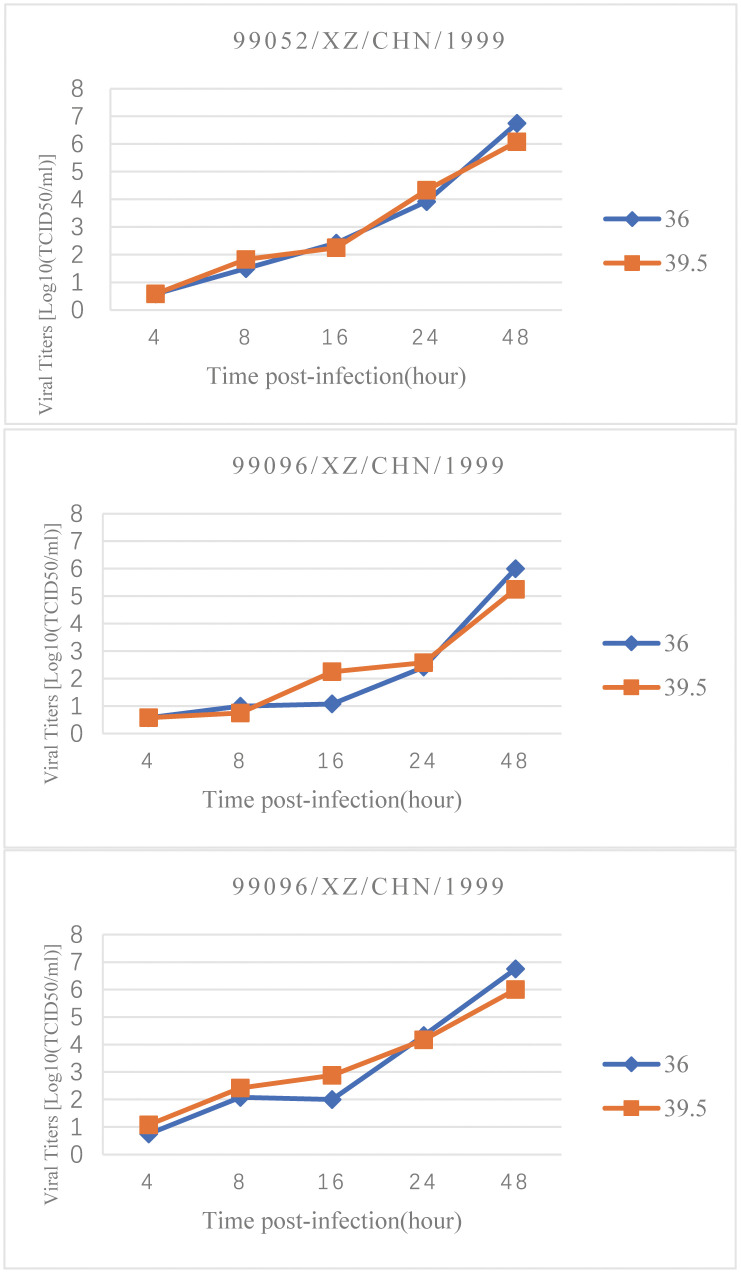
Temperature sensitivity test results showed that the three Tibet EV-B93 strains are all temperature resistant strains. The viral titres of the three EV-B93 strains at different time points are represented in the coordinate system. The blue line represents the growth curve at 36°C and the orange line represents the growth curve at 39.5°C.

Identity plot and bootscanning analyses were used to determine the existence of recombination events between the Chinese EV-B93 strain and the other EV-B prototype strains (Fig 1). Because the three Tibet EV-B93 strains were highly similar, strain XZ99052 was randomly selected as the query strain and analysed, as described above. The nucleotide identity between strain XZ99052 and the prototype strain was higher in the *P1* region, as shown in the Fig 3. Based on BLAST analysis, there are no strains that have particularly high nucleotide identity derived from the query set correlated strongly with respect to the *5UTR*, *P2*, and *3UTR* regions, including the prototype strains. In the *P3* region, a high degree of nucleotide identity (88.8%) was observed with the EV-B107 prototype strain (strain TN94-0349, GenBank Number: AB426609), and the highest similarity of this strain with the EV-B93 strains is observed in the *3D* region (nt positions 6181 and 6961), indicating that recombination occurred during the evolution of the Tibet EV-B93.

### Seroprevalence of EV-B93 in Tibet

VP1 is the outermost part of the enterovirus capsid protein and the major neutralizing determinant. Many enterovirus vaccines and antiviral drugs targeting the VP1 protein have been developed. Neutralization tests to assess the levels of neutralizing antibodies in the population can accurately reflect the immune protection status of the population. A sero-epidemiology study was performed by using 56 sera samples from healthy children in Tibet; the results showed that the neutralizing antibody titres against EV-B93 were generally lower than those against EV-B81 in a previous study [43] in Tibetan children in 2010: titres of <1:8, 1:8–1:64, and >1:64 accounted for 23.21%, 76.78%, and 0%, respectively, and the GMT was 1:9.94 (Table 4). However, the GMT of EV-B81 tested using the same serum samples in Tibet was 1:27. The prevalence of EV-B93 was lower than that of the widely present CV-A16 and EV-A71 strains [44].

**Table 4:** Seroepidemiology of EV-B93 from healthy Tibetan children.

Titres	Shigatse	Lhasa	Total
<1:8	4	9	13 (23.21%)
1:8–1:64	4	39	43 (76.78%)
>1:64	0	0	0
Total	8	48	56

### Tibet EV-B93 showed temperature resistance

Temperature sensitivity is an important characteristic of enteroviruses. More than 2.0 logarithmic differences indicated temperature sensitivity phenotype in temperature sensitivity test. The Tibet EV-B93 was temperature resistant unlike the EV-B106 strains (KS-MGTH90F/XJ/CHN/2011) [9], which showed temperature sensitivity (Fig 3). The polyprotein ORF regions of the three strains were compared, and we found only 13 non-synonymous amino acid substitutions in the polyprotein ORF in all three strains by the full-length genomic sequence analysis. The consistency of the three strains in temperature-sensitivity experiments suggests that the 13 non-synonymous mutations have no conclusive influence on the temperature sensitivity of the strain.

## Discussion

By the end of 2018, only 33 cases of polio were reported worldwide; type 2 and type 3 wild-type polioviruses appear to have been eradicated, and the transmission of type 1 wild-type polioviruses persists in only two countries—Pakistan and Afghanistan [45]. Thus, the AFP cases caused by polioviruses are less than that caused by the non-polio enteroviruses. Many enterovirus serotypes such as EV-A71, E-11, and E-6 have been frequently reported as pathogens in AFP cases, and some recently discovered enteroviruses such as EV-B94, EV-C105 have also been identified as the causative pathogens of AFP, although most of the viruses were isolated from the faeces of the AFP patients [46]. Little research has been carried out on recently discovered enteroviruses, as efforts aimed at discovering new strains have been scarce. The pathogenicity of recently discovered enteroviruses needs further understandings, which generally rely on animal models or cell level experiments. In this study, three EV-B93 strains were identified; the specimens from which the viruses were isolated were collected from AFP patients during surveillance of AFP cases in Tibet, China, in 1999. Only a few EV-B93 strains have been identified in the world, and there are even fewer studies on their aetiology and pathogenicity. The aim of this study was to characterize the Chinese EV-B93 strain and enrich our knowledge of EV-B93.

Very few EV-B93 is reported worldwide. Currently only 15 strains of EV-B93 have been reported worldwide, all of which were isolated from the faeces of the AFP patients—eight from India and the others from other Asian and African countries. There are no reports from Europe and the United States as of 2018. However, this does not necessarily mean that the incidence of EV-B93 is low, probably because most infections do not trigger any symptoms. The EV-B93 sequence obtained from GenBank can be divided into three clusters; three Chinese EV-B93 belong to cluster 2, which has higher nucleotide identity with Pakistan strains.

Point mutation and recombination are two important evolutionary mechanisms of enteroviruses. Many studies on poliovirus have shown that, two genetic characteristics, nucleotide substitutions and recombination, seem to underlie the occurrence of poliomyelitis outbreaks associated with circulating vaccine-derived polioviruses, which shows different characteristics from Sabin strain, such as the capacity for sustained person-to-person transmission, “non-vaccine-like” antigenic properties, higher neurovirulence, replicate at a higher temperature, etc [47]. Therefore, it is particularly important to monitor changes in the biological properties of the enteroviruses, and then study their epidemiology.

The enterovirus genome is constantly evolving due to the influence of human immunity through genetic mutation and gene recombination. Recombination is an important force in the evolution of enteroviruses; thus, the recombinant studies of viruses play an important role in analysing the evolution and genetic diversity of viruses [48–52]. Recombination usually occurs between genomes with high nucleotide identity; for example, the recombination of enteroviruses usually occurs between viruses within the same species [53], thus, co-infection with high sequence identity of viruses in cells is the premise of recombination. Recombination may occur between the same serotypes, and between serotypes with near evolutionary relationships [54–56]. The increase in adaptivity of the virus under selective pressure is conducive to the prevalence of the virus. Continuous genetic variation and genetic recombination lead to new mutations. The emergences of new strains lead to viral outbreaks. The nucleotide and amino acid sequence identity among the three Tibet EV-B93 strains and the prototype strain were 83.2%–83.4% and 96.8%–96.9%, respectively.

The polymerases of enterovirus that are involved in replication lack exonuclease activity; thus, mismatched bases cannot be corrected. This leads to high mutation rates and high self-replication rates, resulting in genetic diversity. The new variants that have caused global outbreaks are representative of such genetic mutations. For example, the transformation of the genotype of CV-A6 epidemic strains caused a large-scale outbreak by the genotype D3, which is more virulent than genotype D2 [57], and the evolutionary branch C4b of EV-A71 disappeared and the evolutionary branch C4a caused outbreak and spread [58, 59]. Recombination usually occurs between genomes with high nucleotide identity. As the non-structural coding region mainly encodes the functional protein of the virus, its mutation is not conducive to the adaptation and survival of the virus. Selection of a mutant sequence that is not conducive to survival is eliminated, and the stable non-structural protein coding region undergoes continuously recombination and evolution to increase whole genome diversity, enabling normal functioning and thereby leading to an adaptive advantage.

In this study, the geometric mean titer (GMT) of the three Tibetan strains of EV-B93 was 1:9.94, which was lower than that of EV-A71 and CV-A16, which are widely prevalent in China. However, the seroprevalence study was performed on a very small sample in this study, which limits the conclusions that can be made about the circulation of EV-B93 in Tibet.

The three Tibetan EV-B93 strains were genetically similar, and showed temperature resistance. In recent years, China has set up an AFP case surveillance system and a hand, foot, and mouth disease surveillance system to detect enteroviruses, mainly studying the cyclical epidemiology, the evolution of pathogenic mechanisms, gene recombination of different serotypes, the development of genetically engineered vaccines and diagnostic reagents. Data on the relationship between different serotypes and clinical symptoms may help to predict the occurrence of related diseases.

The extensive use of molecular analysis to monitor enteroviruses will help scientists better understand the prevalence of enteroviruses. The recently discovered enteroviruses can be isolated from a variety of populations, animal groups, and environment, which provides an important theoretical basis for the detection of the pathogenicity, transmission, circulation, and evolutionary model of the recently discovered enterovirus, thus strengthening the prevention and treatment of the diseases caused by enteroviruses.
